# Comparison of Respiratory Distress Syndrome Amongst Preterm Twins (28-34 Weeks) Born Within and After Two Weeks of Completion of Single Antenatal Corticosteroid Course: a Bidirectional Cohort Study

**DOI:** 10.34763/jmotherandchild.20212504.d-21-00034

**Published:** 2022-04-18

**Authors:** Monisha J Arulalan, Gowri Dorairajan, Nivedita Mondal, Palanivel Chinnakali

**Affiliations:** 1 Department of Obstetrics and Gynecology, Jawaharlal Institute of Postgraduate Medical Education and Research (JIPMER), Puducherry. India; 2 Department of Neonatology, Jawaharlal Institute of Postgraduate Medical Education and Research (JIPMER), Puducherry, India; 3 Department of Community Medicine, Jawaharlal Institute of Postgraduate Medical Education and Research (JIPMER), Puducherry, India

**Keywords:** Antenatal corticosteroids, Preterm twins, Perinatal morbidity, Respiratory distress syndrome, Respiratory morbidity, Steroid-delivery interval

## Abstract

**Background:**

The literature on neonatal outcomes in preterm twins delivered before 34 weeks but within and after 14 days of a single initial steroid course is limited.

**Material and methods:**

This bidirectional (226 prospective and 42 retrospectives) cohort study was performed at a tertiary care teaching hospital in South India. We compared the respiratory distress syndrome and neonatal death amongst preterm twins from 28 to 34 weeks born < 14 days (Group A, n=268) and after 14 days (Group B, n=268) of completion of a single course of antenatal steroids. We used multivariable regression analysis (log-binomial model) to adjust for confounding variables. We generated a propensity-matched score with probit regression to analyse outcomes (respiratory distress and neonatal deaths).

**Results:**

The two groups had significant differences in the distribution of birthweight, gestation period and mode of delivery. On adjusted analysis, the period of gestation below 33 weeks and weight below 1.5 kg had the maximum influence on respiratory and other morbidities, and weight less than 1 kg on neonatal death. [adjusted relative risk (ARR) 26.06, (95%CI=2.37-285.5), p=0.008]. On propensity scoring after matching all these variables, we found an [ARR of 2.0 (95% CI: 1.03-3.88), P=0.017] for neonatal death after 14 days of steroid injection. The ARR for respiratory distress syndrome was 1.13 in those born after 14 days of steroids, though it did not reach statistical significance.

**Conclusion:**

On propensity scoring, the steroid-delivery interval more than 14 days was associated with a significantly increased risk (ARR of 2) of neonatal death.

## Introduction

Antenatal corticosteroids (ACS) are a landmark strategy to reduce respiratory complications among premature neonates and reduce adverse perinatal outcomes. [1, 2] It is recommended for those likely to deliver before 34 weeks with the maximum benefits when delivery occurs within 7 days of steroids [3,4,5,6]. Similar recommendations have been suggested for twin gestation also. Recent metanalysis [7] that included 17 observational studies on preterm twins also concluded favouring the beneficial effects of steroids for preterm twins.

Recommendation for a repeat course of steroids is variable. The Royal College of Obstetricians and Gynaecologists does not advocate its routine usage. The International Federation of Gynaecologists and Obstetricians (FIGO) and the American College of Obstetricians and Gynecologists (ACOG) recommends the steroids if 14 days have passed since the initial dose and the woman is at risk of imminent delivery within 7 days and before 34 weeks. WHO recommends a repeat dose after 7 days of initial steroids, and ACOG also recommends that a rescue dose be considered after 7 days [8]. Recently, authors have reported that repeat doses adversely affect the birth weight and growth [9, 10]. Most evidence on steroid delivery interval and outcomes is a subgroup analysis restricted to within and after 7 days. Few studies [11,12,13] have shown that delivery before 34 weeks and after 14 days of steroids is associated with a significantly increased risk of adverse outcomes. These studies are restricted to singleton pregnancies. The administration of steroids in multiple gestations is mistimed due to its prophylactic use, resulting in a need for a repeat dose [14]. There is a lack of data on short-term outcomes in twin gestation born before 34 weeks but after 14 days of a single course of antenatal steroids. We undertook this bidirectional cohort study amongst twins delivering before 34 weeks to compare the short-term respiratory morbidities among those born within and after14 days of a single course of antenatal steroids.

## Material and methods

### Study design and setting

An observational bidirectional cohort study (226 prospective and 42 retrospectives) was carried out in the Department of Obstetrics and Gynecology, JIPMER; tertiary care teaching hospital in Puducherry, South India.

The Institute Ethics Board approved the study. The work conforms to the provisions of the Declaration of Helsinki. We obtained written informed consent from all women enrolled in the study.

### Participants

All the women with twin gestation who completed a single course of antenatal corticosteroids from April 2018 to November 2019 were followed up and included if they delivered between 28 and 34 weeks of pregnancy.

### Exclusion Criteria

We excluded women of 18 years or less, anomalous fetus, single fetal demise, twin to twin transfusion syndrome, more than one or incomplete course of steroid, pregestational or gestational diabetes on insulin, major placenta previa and women delivering after 34 weeks.

We used the last menstrual period or first-trimester scan in patients with suspected dates to calculate the gestation period. Either betamethasone (two intramuscular doses of 12 mg, 24 hours apart) or dexamethasone (four doses of 6 mg each administered intramuscularly 12 hours apart) was considered a completed course of ACS. We studied the following groups.

Group A: Twins between 28 and 34 weeks born on or before 14 days after completion of the single antenatal corticosteroids course.

Group B: Twins between 28 and 34 weeks born after 14 days of the completed single antenatal corticosteroid course.

We collected case details as per proforma, including antenatal and intrapartum period details and the neonatal outcome. We compared neonatal outcomes in the two groups.

### Outcome

#### Primary

To compare the incidence of respiratory distress syndrome (RDS) in the two groups. We defined RDS as clinical evidence of respiratory distress along with any one of the following: need for continuous positive airway pressure for ventilation, fraction of inspired oxygen >30% or radiological evidence of RDS or administered surfactant (usually if the respiratory distress does not settle by 6 hours after birth).

The **secondary** outcome was composite morbidity, defined as the occurrence of one or more ischemic necrotising enterocolitis, hypoxic-ischemic encephalopathy, intraventricular haemorrhage or neonatal death. Combined respiratory morbidity (CRM) included transient tachypnoea, apnea and pneumonia.

#### Sample size

In twins delivering before 34 weeks but after 14 days of completion of prophylactic steroids, the incidence of RDS is likely to be the same as that of those not exposed to steroids, which is 51%. Assuming that it will be 33% in those delivering within the optimal duration of prophylactic ACS [15], the sample size needed was 133 (Kelsey and Fleiss with continuity correction) in each group to achieve a power of 80% and an alpha error of 5%. Therefore, we studied 134 women in both groups (268 total). We considered each mother as a single unit. We sampled all eligible women consecutively in the study period.

To complete the sample size, we included data from the records of 42 twin pregnancies who had preterm delivery before the study period.

We followed up with the neonates till discharge from the hospital.

### Statistical Analysis

We used Statistical Package for Social Sciences (SPSS 20.0 for Windows to analyse the data). The confounding variables identified were chorionicity, comorbidity of gestational hypertension and effect modifiers as the gestation period, birthweight, twin birth order (first or second).

We checked the normality distribution of continuous data by the Kolmogorov Smirnov test. These parameters were compared for two groups by Student's t-tests (if normal) and mean ± SD presented their descriptive data. The Mann Whitney test was used for skewed and other ordinal data and their descriptive data presented by median/interquartile range. We analysed classified or categorical data as frequencies and proportions and analysed its association with the groups using the Chi-square test or Fisher's exact test, whichever is applicable. We compared the outcomes in the two groups. We used the Student t-test for continuous and the Chi-square test for categorical variables respectively to determine the significance for any difference. P-value <0.05 was taken as significant. We carried out multivariable regression analysis (log-binomial model) to adjust for confounding variables and effect modifiers on the primary outcome in the two study groups. We calculated adjusted relative risk (ARR) with a 95% confidence interval from the model. We did propensity score matching between the groups (≤7 days and >7 days); (≤14 days and >14 days) based on the following variables: age, mode of conception, type of antenatal corticosteroid, hypertensive diseases, mode of delivery, gestational age at delivery and birth weight. We used probit regression to generate the propensity score. We calculated standardised mean differences to assess the balance after matching. We used the matched datasets to analyse outcomes (respiratory distress and neonatal deaths) in regression models.

## Results

**Figure 1:**
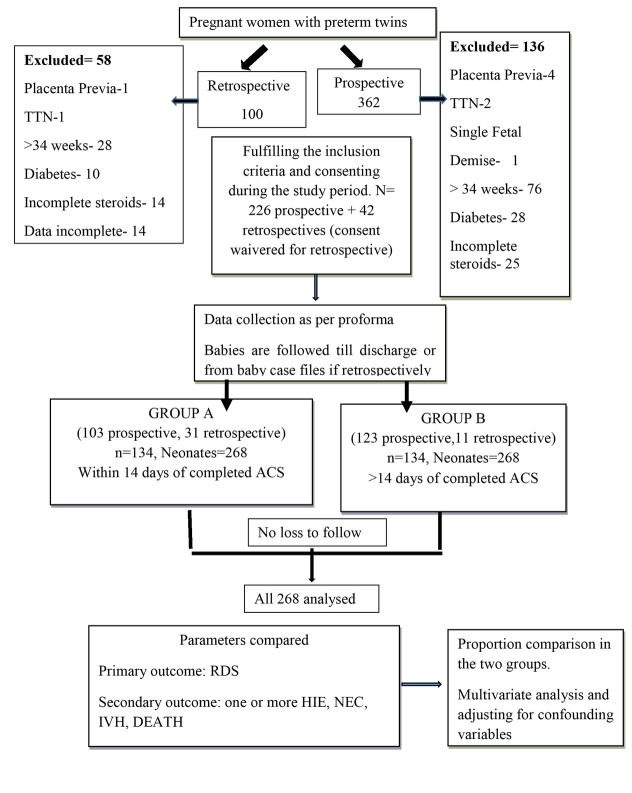
Flow chart of the study.

There were 32,877 deliveries in the study period. There were 653 women with twin gestation (1.1%). Three hundred and sixty-two amongst them were preterm (55.4% prematurity rate). We prospectively recruited 226 women who fulfilled the inclusion criteria during the study period. We screened 100 records of the previous six months, and 42 were enrolled retrospectively (Figure1). The mean age of the study cohort was 27.5 years. The proportion of women above 35 years was 9%. Three-fourths of the study cohort were primigravida. Group B had a significantly higher proportion of women over 35 years and women who conceived with artificial reproductive techniques (ART). Group A had a significantly higher proportion of women delivering before 32 weeks, women with preterm rupture of membranes, threatened preterm labour, neonates with birthweight less than 1.5 kg and significantly lower cesarean section deliveries (Table1). The other variables like the education status, mean age, parity and chorionicity were comparable in the two groups. One hundred and twenty-eight women (256 neonates) in group A and 94 in (188 neonates) in group B were in spontaneous preterm labour (X^2^=59.4, p<0.00001). Others received steroids prophylactically, anticipating preterm labour because of twin gestation. Around one-third of those in preterm labour received tocolysis. Those who were more than 32 weeks with monochorionic placentation (n=17, 5%), with PPROM (n=80, 30%), and hypertension (n=58,22%) or discordance did not receive tocolysis.

**Table1:** Maternal demographic and obstetrics variables in the two groups

Baseline maternal characteristics		<14 Days Group A	>14 Days Group B	
		(N=134)	(N=134)	
**AGE years**	19-25 years	62(46.27%)	47(35.07%)	0.017^a^
	26-34 years	66(49.25%)	69(51.49%)	(𝑥^2^ = 8.13)
	≥35 years	6(4.48%)	18(13.43%)	
**EDUCATION**	Illiterate	2(1.4%)	1(0.7%)	0.531 (𝑥^2^ =7.05)
	Primary	16(11.9%)	15(11.5%)	
	Middle	53(39.5%)	50(37.3%)	
	Higher	49 (36.5%)	49(36.6%)	
	Graduation	14 (10.4%)	19(14.2%)	
**PARITY**	Primigravida	101(75.67%)	98 (73.13%)	0.675 (𝑥^2^ =0.17)
	Multigravida	33 (24.33%)	36 (26.87%)	
**TYPE OF STEROID**	Betamethasone	246	196	<0.0001 ^a^ (𝑥^2^ = 32.25)
	Dexamethasone	22	72	
**MODE OF CONCEPTION**	*SPONTANEOUS (n=141)*	83(61.94%)	58(43.28%)	0.002* (𝑥^2^ = 17.25)
	*ASSISTED (n=127)*	51 (40.2%)	76 (59.8%)	
**CHORIONICITY**	Monochorionic (n=58)	26(19.40%)	32(23.88%)	0.373 (𝑥^2^ =0.79)
	Dichorionic (n=210)	108(80.60%)	102(76.12%)	
**COMORBIDITY**	HTD^b^ (n=58)	26(19.40%)	32(23.88%)	0.013 ^a^ 𝑥^2^ =(8.88)
**Preterm premature rupture of membranes**		63(23.5%)	17(6.3%)	<0.00001 ^a^ (𝑥^2^ = 31.5)
**GESTATIONAL AGE at DELIVERY**	Mean ± SD^c^	31.5(±2.09)	32.6 (± 1.75)	<0.001 ^a^
	28-29^+6^(n=52)	38(28.36%)	14(10.45%)	<.001 ^a^ (𝑥^2^ =40.716)
	30-32^+6^(n=68)	39(29.10%)	29(21.64%)	
	33-34 (n=148)	57(42.54%)	91(67.91%)	
**MODE OF DELIVERY**	Caesarean (n=232)	95(35.4%)	137(51.1%)	<0.001 ^a^ (𝑥^2^ =13.40)
	Vaginal (n=304)	173(64.6%)	131(48.9%)	

a- significant. b- Hypertensive disorders. c-SD= standard deviation.

There were no stillbirths. We found that 100% of the babies born <30 weeks had RDS irrespective of steroid delivery interval. Similarly, 100% of babies born <1 kg had RDS regardless of the steroid delivery interval.

Univariate analysis showed that lower periods of gestation, lower birth weight and vaginal delivery were associated with a significantly higher association with primary and secondary outcomes.

The birth order and type of chorionicity did not influence the occurrence of RDS or CRM. On adjusted analysis (Table 2), the risk of primary outcome was highest with lower birth weights and with a period of gestation less than 30 weeks (ARR 1.8, 95% CI 1.17-2.8, p=0.008). It remained significantly higher till 32^+6^weeks (ARR 1.76, 95% CI=1.1-2.5, p=0.004). The adjusted risk of neonatal death was highest among birth weight less than 1 kg. RDS was similar in both the groups amongst those born between 33 and 34 weeks and came down to 21.5 and 20.3% for groups A and B, respectively.

**Table 2:** Multivariable regression analysis of factors influencing respiratory distress syndrome and neonatal deaths

Variable (Reference Group)		RDS (respiratory distress syndrome)		Neonatal Deaths	
		ARR^a^ (95% CI^b^)	P-value	ARR^a^ (95% CI^b^)	P-value
Steroid Group B (Ref-GroupA)		1.09 (0.84-1.4)	0.510	1.06 (0.620-1.8)	0.810
Twin Order 2 (Ref-Twin order1)		1.012 (0.790-1.2)	0.921	1.20 (0.72-2.00)	0.484
Twin Dichorionic (Ref-monochrionic)		1.01 (0.744-1.3)	0.920	1.11 (0.53- 2.30)	0.778
Period of Gestation(weeks) (Ref-33-33^+6^weeks)	28-29^+6^	1.84 (1.17-2.8)	0.008^c^	1.75 (0.48-6.3)	0.392
	30-32^+6^	1.75 (1.1-2.5)	0.004^c^	1.49 (0.405-5.52)	0.545
Caesarean Delivery (Ref-Vaginal Delivery) -		1.28 (0.95-1.7)	0.102	0.987 (0.454-2.14)	0.975
Birth Weight (Ref- ≥ 2kg)	<1 kg	5.60 (2.7-11.2)	0.0011 ^c^	26.06 (2.37-285.5)	0.008 ^c^
	1-1.49 kg	5.23 (2.8-9.6)	0.0001 ^c^	6.30 (0.619-64.09)	0.120
	1.5-1.99 kg	2.8 (1.5-5)	0.001 ^c^	0.716 (0.434-11.82)	0.816
1 min Apgar< (Ref- I min Apgar score>7)				2.46 (0.81-7.41)	0.110
Sex Male (Ref- Female)				1.07 (0.62-1.82)	0.802

a- ARR= adjusted relative risk, b-CI=confidence interval. c- significant

On propensity-matched scoring (Table 3), we found that the more than 14 days group had a significantly higher risk of neonatal death (Adjusted relative risk of 2.0 (95% CI: 1.03-3.88), P=0.017). The RDS was more in the >14-day group with ARR of 1.13, though it did not reach significance. ARR for RDS was 1.04, and neonatal death was 1.85 amongst those born after 7 days compared to those born on or before 7 days of steroids, though it did not reach statistical significance.

**Table 3:** Propensity-matched scoring

Outcome	Grouping	Matched Pairs	Adjusted Relative Risk (95% CI^a^)	P-value
**RDS^b^**	<14 vs >14 days	Grp A (n=180)	ref	
		Grp B (n=180)	1.13 (0.93-1.33)	0.231
**ND^c^**	<14 vs >14 days	Grp A (n=180)	ref	
		Grp B (n=180)	2.0 (1.03-3.88)	0.017
**RDS**	<7 vs >7 days	Grp A (n=161)	ref	
		Grp B (n=161)	1.04 (0.84-1.28)	0.722
**ND**	<7 vs >7 days	Grp A (n=161)	ref	
		Grp B (n=161)	1.85 (0.97-3.50)	0.052

a- CI: confidence interval. b-RDS: respiratory Distress Syndrome. c- ND: Neonatal death.

## Discussion

We compared the RDS and other outcomes between those delivering before and after 14 days of a single course of completed steroids amongst preterm twins delivering from 28 to 34 weeks. We studied 536 neonates (268 in each arm). The groups in our study had significant differences in the distribution of confounding variables and effect modifiers.

The higher distribution of lower gestation period, premature rupture of membranes, and lower birth weight in the < 14 days group are natural. The proportion of older women and those who conceived with assisted reproductive techniques, and hence hypertensive disorders and cesarean delivery, were significantly higher in the more than 14-day group. The number of women in preterm labour was more in the < 14-day group. The proportion of women who received steroids prophylactically was more in the > 14-day group. There is a tendency to give prophylactic ACS to women who have conceived twins with ART at 30 weeks or so, fearing possible preterm labour. Rottenstreich et al.· [14] also observed that only 20% of twins deliver within the optimum window of 7 days of the initial course of steroids. The steroid administration is mistimed in twin gestations. We included all these factors in the multivariable regression analysis.

Our study found that respiratory distress had the highest occurrence in those with lower gestations and birth weight. Many authors have reported similar observations [16, 17, 18, 19, 20].

The Cochrane review on the use of antenatal corticosteroids for preterm birth done in 2017 by Robert et al. [1] included 30 studies. However, the majority were on singletons, and only 12 trials, also had twins. The authors found that single-dose ACS reduces respiratory and other morbidities even in twins. Still, this metanalysis could not establish the optimal time interval since the first steroid course for preterm twins. The updated review in 2020 [2] also recommends more studies on focussed groups like preterm twins.

Very few authors have studied the influence of interval from steroid to delivery on the neonatal respiratory and other morbidities of preterm twin neonates delivering before 34 weeks.

Palas et al.· [21] analysed women with twins delivered from 24 to 31 weeks from the Epiphage 2 cohort study and enrolled 750 twins. They observed that ACS lowered the in-hospital mortality only in the <7 days group. They further found that there was no significant difference in the outcomes between single completed and repeated doses. Nearly 64% of their study population had a caesarean delivery.

In the study by Kuk et al. [15], the authors compared the outcome based on <2, 2-7 and >7 days intervals from single prophylactic steroid to delivery in preterm twins. Though they studied 234 mothers spanned over 16 years, only 114 and 66 neonates were analysed in the less than and more than 7 days group, respectively. Like our study, 75% of their study population were primigravida, and 45% conceived using artificial reproductive techniques. They observed that RDS is reduced significantly in the 2-7 interval group. In their study, 33.3% in the less than 7 days and 39.4% in the more than 7-day group had RDS. It is important to note that 76% of their women in less than 7 days and 88% in the more than 7 days had a caesarean delivery, whereas, in our study, 35% in less than 14 days and 51% in the more than 14 days had a cesarean delivery.

Kaczynska et al. did a retrospective study over 9 years [22]. They studied respiratory outcomes amongst preterm twins delivered before 34 weeks based on the interval from steroid to delivery of lesser (99 neonates) or more than 7 days (112 neonates). Their groups were different only in body mass index. They did not exclude twin to twin transfusion syndrome or diabetes. The RDS rate was 32 and 39%, respectively, in the two groups. The authors observed that ACS did not reduce the RDS significantly in the group delivered within 7 days, but the CRM was lower. Their cesarean section delivery rate was 100% in the <7-day group and 89% in the more than 7-day group.

The caesarean section rate among twins in various studies was found to be very high varying from 56 to 80% [15,21,23,24].

The currently available evidence in the literature regarding mode of delivery in preterm twin gestation does not favour cesarean section [25]. As a policy in our hospital, we encourage vaginal delivery, especially when the women are in spontaneous preterm labour with the first twin in vertex presentation. Our hospital is a tertiary care hospital with 1,500 deliveries a month with high turnover and load on neonatal intensive care unit. The salvageability of babies below 30 weeks or with <1.2 kg is only 50%. In younger women with extreme preterm twins and first twin in vertex presentation, vaginal delivery is encouraged.

Most of these studies compared outcomes among the preterm neonates born before and after 7 days and not after 14 days.

On the propensity score-matched data, our study found that the risk of neonatal death was significantly higher in the more than the 14-day group with an ARR of 2. The risk of neonatal death (ARR-1.85) and RDS (ARR-1.04) was higher though not significant in the 7-to-14-day group compared to the < 7-day group. The RDS was more in the >14-day group with an ARR of 1.13, though it did not reach significance.

Few authors have studied the benefits of steroids in the extended period beyond 7 days of the initial course of steroids.

Wilms et al. [11] observed that the beneficial effect reduces after 7 days of steroids. They found the odds of adverse respiratory outcome and risk of intubation to be 2.3 in those delivering between 8 to 14 days and 5.6 when born 15 to 21 days after steroid course. A similar observation was made by Ring and colleagues [12]. Battarbee [13] observed that the risk of RDS increased 2.34 times in those delivering before 34 weeks but after 14 days of steroids. These authors studied only singleton pregnancies.

In the retrospective cohort study, Bibbo et al. [24] compared the outcomes among twins delivering before 34 weeks receiving rescue dose after 14 days of initial steroids (42 neonates) with those not receiving the rescue dose (88 neonates). They observed that though the RDS was not different, the respiratory performance was significantly better in the rescue dose group. In the double-blind, randomised control trial by McEvoy et al. [26], the authors showed significantly increased respiratory compliance in women receiving a repeat course of steroids after 14 days. They had included 12 twin gestations in the rescue arm and 16 in the placebo arm. In the randomised trial by Garite et al. [27], there were 67 twins in the rescue group and 74 twin pregnancies in the placebo group. The authors had shown a significant reduction in composite morbidities and improvement in respiratory morbidities among those delivering before 33 weeks and receiving rescue steroids after 14 days of the initial dose compared to those in the placebo group.

Thus, very few authors have studied twins' outcomes before 34 weeks but after 14 days of the initial steroid therapy. As reviewed by Dehaene et al. [28], most of the studies in the literature on neonatal outcomes based on a steroid-delivery interval are observational with subgroup analysis and not targeted trials. Our study enrolled 268 neonates in both groups at recruitment. And so, there was no dilution of the sample size. Our study found that deliveries of twins delivered before 34 weeks but after 14 days of a single course of antenatal steroids have a significantly higher risk of neonatal death. The RDS and neonatal mortality in the more than the 7-day group compared to the within the 7-day group was higher, though it was not statistically significant.

### Interpretation of our study

The risk of neonatal death significantly increases twofold in those preterm twins born before 34 weeks after 14 days of exposure to steroids. The risk of death and RDS was marginally but not significantly increased in the twins born after 7 days of steroids compared to those born on or before 7 days of completion of the initial course of steroids. The results of our study endorse the need to repeat steroids after 14 days of completion of steroids in preterm twin gestations who are at risk of delivery within 34 weeks in cases where the initial dose was mistimed. We recommend randomised trials to study the benefit of repeat steroids after 14 days of the first course of steroids among the group of preterm twins delivering before 34 weeks of gestation.

## Strength and Limitations

The strength of our study is that it is on a homogenous population of preterm twins (28 to 34 weeks) with a good sample size of 268 neonates in each group. Many antenatal characters that influence perinatal outcome were studied and analysed for adjusted risk analysis, including birth order and chorionicity. We followed strict criteria for defining the RDS and a uniform standard treatment protocol for all cases.

We had 226 prospective and only 42 retrospective cohorts. We did propensity-matched scoring to adjust for the unequal distribution of confounders and effect modifiers. Our institute is a tertiary care institute of national importance with a state-of-the-art neonatal intensive care facility with high salvageability and a reasonable prognosis of neonates born after 30 weeks or more than 1.2 kg. The neonatal salvageability cannot be generalised across various hospitals in India with limited resources. So, to conclude, our study, we found that the gestation period less than 32^+6^ weeks and birthweights below 1.5 kg have the maximum influence on the occurrence of respiratory morbidity among preterm twins delivering before 34 weeks and who received a single course of ACS. Birth weight less than 1 kg has the most substantial influence on neonatal death. We further found on propensity-matched scoring that the risk of neonatal death increases significantly after 14 days of the steroid injection amongst preterm twins born between 28 and 34 weeks of pregnancy.

## Key Points

- Amongst preterm (28–34 weeks) twin births, we observed that gestational age lower than 32^+6^ and birthweights less than 1.5 kg had the maximum risk of respiratory morbidity despite antenatal steroids.
- Birth weight less than 1 kg has the highest risk of neonatal mortality despite antenatal steroids and needs to be an essential point while counselling.
- We recommend strategies to reduce twin conception in ART, as we found that 47% of the cases followed ART.
- We recommend studies on predicting and preventing preterm labour amongst twin gestation to reduce the adverse outcomes as nearly 55 of twins screened had preterm delivery.
- On propensity-matched scoring, the RDS and neonatal death was marginally but not significantly higher in the more than 7 day-group compared to those delivered within 7 days and significantly higher for neonatal death after 14 days of steroid delivery interval.
- It may be prudent to give a second dose of steroids after 14 days of initial course if delivery is imminent before 34 weeks in preterm twins to reduce the neonatal death rates.
